# Multimodal Simulation of a Novel Device for a Safe and Effective External Ventricular Drain Placement

**DOI:** 10.3389/fnins.2021.690705

**Published:** 2021-06-14

**Authors:** Giuseppe Emmanuele Umana, Gianluca Scalia, Kaan Yagmurlu, Rosalia Mineo, Simone Di Bella, Matteo Giunta, Angelo Spitaleri, Rosario Maugeri, Francesca Graziano, Marco Fricia, Giovanni Federico Nicoletti, Santino Ottavio Tomasi, Giuseppe Raudino, Bipin Chaurasia, Gianluca Bellocchi, Maurizio Salvati, Domenico Gerardo Iacopino, Salvatore Cicero, Massimiliano Visocchi, Lidia Strigari

**Affiliations:** ^1^Department of Neurosurgery, Trauma Center, Gamma Knife Center, Cannizzaro Hospital, Catania, Italy; ^2^Department of Neurosurgery, Highly Specialized Hospital and of National Importance “Garibaldi,” Catania, Italy; ^3^Department of Neurological Surgery, University of Virginia, Charlottesville, VA, United States; ^4^MT Ortho Srl, Aci Sant’Antonio, Catania, Italy; ^5^DICAR, University of Catania, Catania, Italy; ^6^Department of Experimental Biomedicine and Clinical Neurosciences, School of Medicine, Postgraduate Residency Program in Neurological Surgery, Neurosurgical Clinic, AOUP “Paolo Giaccone,” Palermo, Italy; ^7^Department of Neurosurgery, Christian-Doppler-Klinik, Paracelsus Private Medical University, Salzburg, Austria; ^8^Department of Neurosurgery, Humanitas University, Catania, Italy; ^9^Department of Neurosurgery, Neurosurgery Clinic, Birgunj, Nepal; ^10^Department of Otorhinolaryngology, San Camillo Forlanini Hospital, Rome, Italy; ^11^Department of Neurosurgery, Policlinico Tor Vergata, Rome, Italy; ^12^Craniovertebral Junction Operative Unit, Master CVJ Surgical Approach Research Center, Institute of Neurosurgery, Policlinic “A. Gemelli”, Catholic University, Rome, Italy; ^13^Department of Medical Physics, IRCCS University Hospital of Bologna, Bologna, Italy

**Keywords:** hydrocephalus, shunt, ventricle, cerebral hypertension, ventricular catheter

## Abstract

**Background:**

External ventricular drain (EVD) placement is mandatory for several pathologies. The misplacement rate of the EVD varies widely in literature, ranging from 12.3 to 60%. The purpose of this simulation study is to provide preliminary data about the possibility of increasing the safety of one of the most common life-saving procedures in neurosurgery by testing a new device for EVD placement.

**Methods:**

We used a novel guide for positioning the ventricular catheter (patent RM2014A000376). The trajectory was assessed using 25 anonymized head CT scans. The data sets were used to conduct three-dimensional computer-based and combined navigation and augmented reality-based simulations using plaster models. The data set inclusion criteria were volumetric head CT scan, without midline shift, of patients older than 18. Evans’ index was used to quantify the ventricle’s size. We excluded patients with slit ventricles, midline shift, skull fractures, or complex skull malformations. The proximal end of the device was tested on the cadaver.

**Results:**

The cadaveric tests proved that a surgeon could use the device without any external help. The multimodal simulation showed Kakarla grade 1 in all cases but one (grade 2) on both sides, after right and left EVD placement. The mean Evans’ index was 0.28. The geometric principles that explain the device’s efficacy can be summarized by studying the properties of circumference and chord. The contact occurs, for each section considered, at the extreme points of the chord. Its axis, perpendicular to the plane tangent to the spherical surface at the entry point, corresponds to the direction of entry of the catheter guided by the instrument.

**Conclusion:**

According to our multimodal simulation on cadavers, 3D computer-based simulation, 3D plaster modeling, 3D neuronavigation, and augmented reality, the device promises to offer safer and effective EVD placement. Further validation in future clinical studies is recommended.

## Introduction

External ventricular drain (EVD) placement is mandatory for several pathologies, both in emergency and elective surgery. It allows reducing life-threatening intracranial hypertension, thus rapidly improving the patient’s clinical conditions and sometimes saving the patient’s life. The misplacement rate of the ventricular catheters reported in the literature varies widely, ranging from 12.3 to 60% (Saladino et al., 2009; Toma et al., 2009; Park et al., 2011; Bergdal et al., 2013; Chai et al., 2013; Rehman et al., 2013; Phillips et al., 2014), with possible serious complications (e.g., cerebral hematomas, neurological deficits, and seizures) (Woernle et al., 2011; Rosenbaum et al., 2013). This misplacement rate has been considered as the baseline for our results, and we aimed to obtain a lower misplacement rate in a simulation setting. The misplacement rates reported in literature are high, probably due to the use of EVD also in patients affected by midline shift or slit ventricles and in procedures performed by non-neurosurgeons in remote areas with logistic difficulties. Numerous studies have already been published about the reduction of complication after EVD, which is currently a freehand procedure, performed only according to anatomical landmarks that help to introduce the catheter perpendicularly to the burr hole; the perpendicularity is a critical element for the correct positioning of the catheter (Gil et al., 2002; Shimizu et al., 2004; Lee et al., 2008, 2020; Mahan et al., 2013; Patil et al., 2013; Jakola et al., 2014; Siesjö, 2014; Shtaya et al., 2018; Thomale et al., 2018; Wilson et al., 2018; Fuller et al., 2020; Park et al., 2020; Sun et al., 2020; Amoo et al., 2021; Cabrilo et al., 2021; Pishjoo et al., 2021).

One study reported the results in 17 patients, using a guide that allows obtaining perpendicularity (Ghajar, 1985): this study reported obtaining cerebrospinal fluid (CSF) on the first attempt, providing initial evidence of the strong correlation between the perpendicular trajectory offered by their device and the correct positioning of the catheter. In 11 out of 17 patients (64%), the correct positioning at the level of the ipsilateral anterior frontal horn of the ventricle was confirmed by intraoperative fluoroscopy and postoperative CT scan. However, this guide was not suitable for neuronavigation, a useful tool when midline shift is present.

The development of new devices is one of the purest forms of surgical research, but it is rare and challenging, since most of the technical discoveries have already been reported. We highlight the experimental nature of this study, in which we used merged technologies to assess the accuracy and validate the trajectory offered by the device, which allows to immediately obtain the perpendicularity required and, indicated in literature, for correct EVD placement.

The purpose of this study is to provide preliminary data about the possibility of increasing the safety of one of the most common life-saving procedures in neurosurgery by testing a new device for EVD placement.

## Materials and Methods

We used a novel guide for positioning the ventricular catheter (patent RM2014A000376), which is neither invasive nor implanted on the patient, and it does not modify the standard surgical protocol for EVD placement. We used a cadaveric specimen, already used for a cadaver lab during the “First European advanced course on surgical techniques for the obstructive sleep apnea-hypopnea syndrome” in Rome, to develop the device’s proximal end, which is the key: it is fixed to the skull, it does not go beyond the dura mater, and, above all, it has a perpendicular trajectory. Several bilateral, precoronal, and prefrontal tests were performed to improve the device’s insertion into the burr hole. The cadaveric specimen was not used to assess catheter trajectory. The entry points were precoronal: 7–11 cm from the nasion and 3 cm from the midline.

The trajectory was assessed using 25 anonymized head CT scans of patients with a mean Evans’s index of 0.28. The data sets were used to conduct three-dimensional computer-based and augmented reality-based simulations. Data set inclusion criteria were volumetric head CT scan without midline shift of patients older than 18. Evans’ index was used to quantify the ventricle’s size. We excluded patients with slit ventricles, midline shift, skull fractures, or complex skull malformations.

### Methodologic Highlights

I. Precoronaric burr hole, 11 cm from the nasion and 5–3 cm from midline and dura mater incision.
II. Internal bone spur removal if the bone is very thin, <8 mm.
III. Insert the device inside the burr hole; associate the navigation reference if midline shift is present.
IV. Insert the catheter inside the device, remove the mandrel, and remove the device.
V. Catheter tunneling.

### Standard Procedure

**FIGURE 1:**
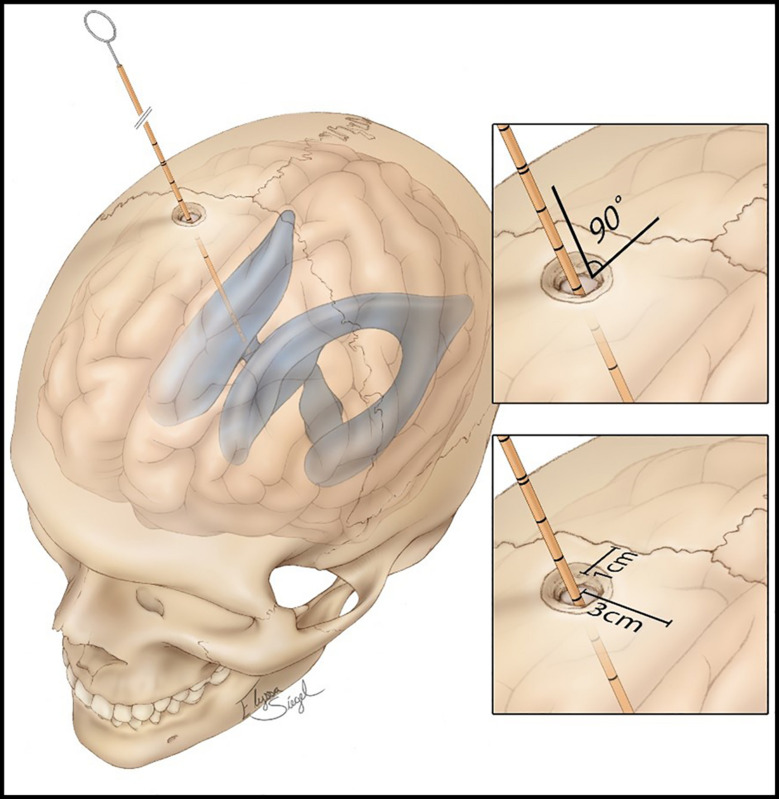
Standard procedure of external ventricular drain (EVD) placement, showing the distances from the coronal suture, midline, and the angle of 90° of the catheter, aiming to perpendicularity. Source: https://assets.neurosurgicalatlas.com/volumes/ATLAS/1-PRINCIPLES_OF_CRANIAL_SURGERY/16-External_ Ventricular_Drain/PCS_ExtIntraventDrain_03.jpg (last accessed: March 30, 2021).

• Step 1: In the standard freehand procedure, the skin incision can be either linear or curvilinear.
• Step 2: A burr hole is placed 10–11 cm from the nasion and 2.5–3 cm on the right of the midline. The burr hole is performed with a 14-mm perforator (Figure 1).
• Step 3: The dura mater is coagulated and cut with a no. 11 blade, and a small corticectomy is performed.
• Step 4: The catheter is introduced for 6 cm from the external cortical bone of the skull, looking for the perpendicularity (90°) compared with the outer skull, having as reference the mid-pupillary line, the external acoustic meatus, and the medial canthus; these are the landmarks for directing the tip of the catheter toward the ipsilateral Monro foramen.
• Step 5: Once the catheter is inserted and CSF is obtained, the catheter is tunneled and connected to the external drainage system.

### Device Description

**FIGURE 2:**
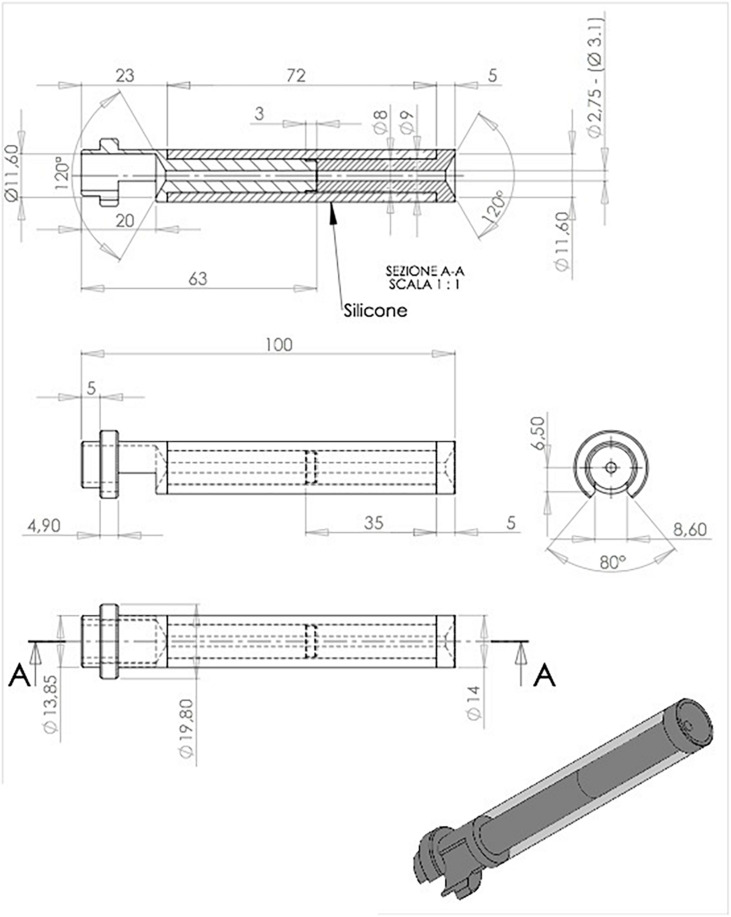
External ventricular drain (EVD) device, detailed illustration.

**FIGURE 3:**
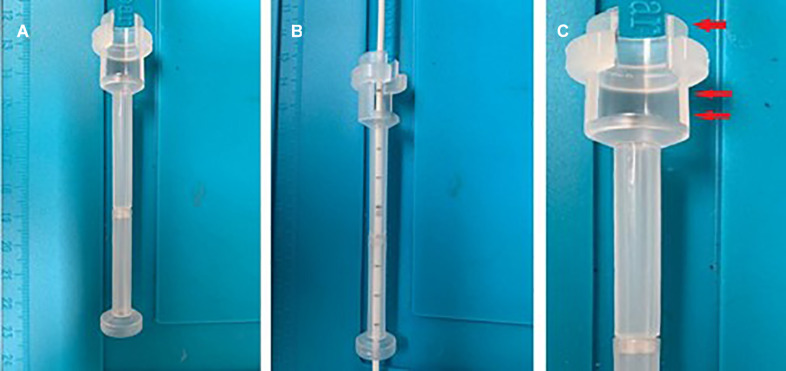
The device is semitransparent, allowing catheter control during insertion **(A,B)**. The catheter’s proximal end (arrow), which helps to fix the device to the skull with the correct trajectory. The proximal end also presents a window to clamp the catheter before removing the mandrel (double arrows) **(C)**.

The device’s peculiarity (Figure 2) is identifiable in the bone support base, which allows the fast and non-invasive achievement of the correct trajectory (perpendicular to the curvature of the skull). The distal cylindrical component maintains the trajectory during the insertion and allows interfacing with the neuronavigation, using the navigated stylet or universal reference systems of the common brands. The device can be sterilized multiple times, saving costs. It is semitransparent, allowing to visualize the number indicating the catheter’s length, thus controlling the depth of insertion (Figure 3).

### Simulation

#### 3D Computer-Based Simulation

Twenty-five anonymized head CT scan data sets were uploaded, and 3D head models were obtained. The skull and ventricles were reconstructed. The 3D rendering of the ventricles was highlighted and visualized together with the skull, removing the brain. A 3D representation of the device was created and positioned in Kocher’s point, along its offset and directed proximally, toward the lateral ventricles. The device was positioned perpendicular to the skull by imposing the flat part of the device’s proximal end on the skull’s curvature.

#### 3D Computer-Based Simulation Protocol

**FIGURE 4:**
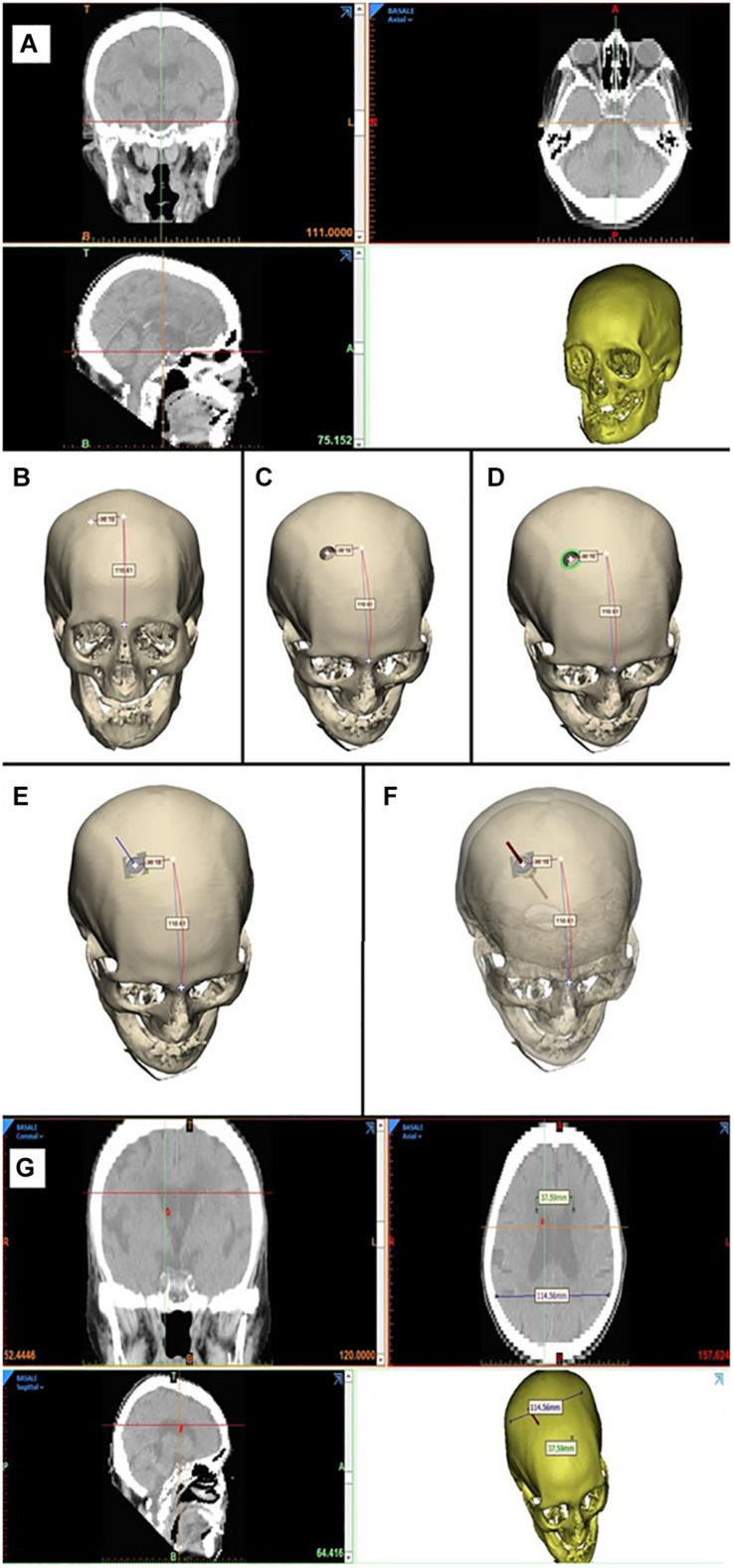
3D computer-based simulation **(A)**, showing the entry point definition based on anatomical landmarks, to both the right and left, Evans’ evaluation, simulation of the catheter placement **(B–F)**, bilaterally obtaining grade A positioning **(G)**.

This protocol was designed to virtually simulate the EVD procedure performed with the aid of the novel device in the most realistic way. This device allows the catheter’s insertion in a guided way; mainly, it ensures the catheter to be inserted perpendicular to the skull’s surface, providing a higher success rate. This simulation can be summarized in seven phases:

• Phase 1: Reconstruction of the 3D model. The 3D model of the patient’s bone is created using the Mimics software (Materialize, Leuven Belgium) from the CT images data set (Figure 4A).
• Phase 2: Identification of the catheter entry point. The 3D model just obtained is imported into the Materialize^®^ 3-Matic software. In this virtual-like environment, using a measuring instrument along curved surfaces, we move 100/110 mm, starting from the glabella, along the sagittal plane, and then 30 mm along the coronal plane, thus identifying the entry point of the catheter (Figure 4B).
• Phase 3: Simulation of the burr hole. Performing a Boolean operation of subtraction between solids, the first surgical phase of the external ventricular drain (EDV) placement, represented by the burr hole, is simulated by subtracting a cylinder with a diameter of 14 mm from the 3D model of the skull (Figure 4C).
• Phase 4: Identification of the contact surface. At this point, the surgical procedure involves the positioning of the device inside the hole, orthogonally to the contact surface. To virtually simulate this surgical phase, the instrument/skull contact surface ensemble was isolated, consisting of a crown with an external diameter of 20 mm (coinciding with the outer diameter of the instrument in contact with the skull) and an internal diameter of 14 mm (inner diameter of the burr hole) (Figure 4D).
• Phase 5: Identification of the normal to the contact surface. The plane that best fits the newly isolated surface is then identified. The normal to this plane will specify the device’s direction of insertion (Figure 4E).
• Phase 6: Catheter entry simulation. This phase allows the virtual simulation of the catheter’s insertion. A cylindrical solid with a diameter of 2.7 mm is created, and it is translated along the direction normal to the plane for a length of 60 mm (Figure 4F).
• Phase 7: Verification of correct catheter placement. The cylinder thus positioned is then imported into the Mimics software, where it can be viewed on the patient’s CT images. In this way, it is possible to verify that the cylinder, simulating the catheter, reaches the ipsilateral lateral ventricle (Figure 4G).

#### Modeling

**FIGURE 5:**
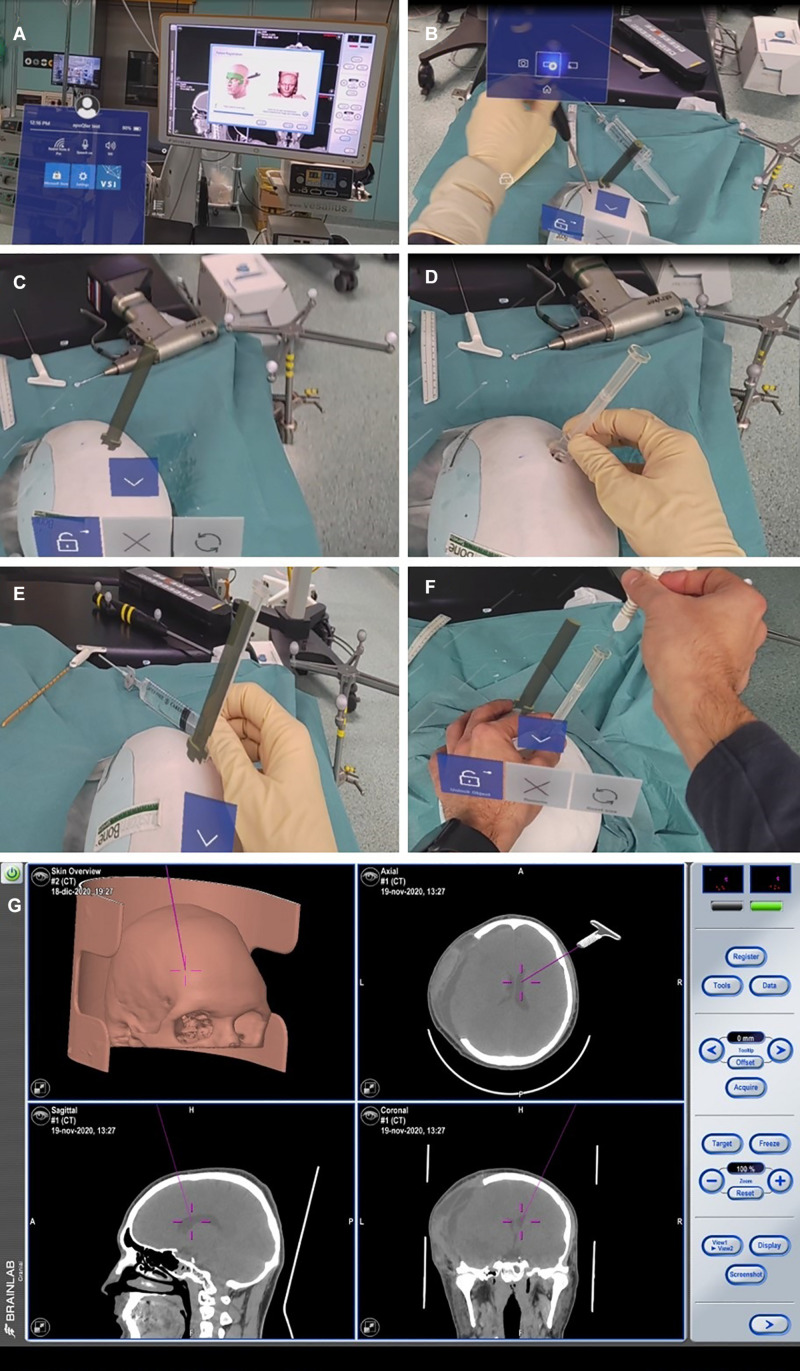
Finceramica plaster model navigation after merging the patient’s CT scan (already treated for post-traumatic craniotomy, but with no midline shift). **(A,B)** Superficial tracing for navigation registration (BrainLab) of the plaster model. **(C)** Entry point definition with a holographic representation of the device. **(D)** Positioning of the real device on the defined entry point. **(E)** Overlap of the holographic device with the real one, to match the entry point. **(F)** Introduction of the navigated stylet inside the device. **(G)** Its representation with the standard navigation.

Twenty-five plaster models of the patients’ anatomy were created (Fin-Ceramica, Faenza, Italy) and used for combined 3D neuronavigation and augmented reality-based navigation (Figure 5). These plaster models are usually used for cranioplasty planning; thus, their accuracy to the patient’s anatomy is the highest available.

#### Augmented Reality-Based Simulation

**FIGURE 6:**
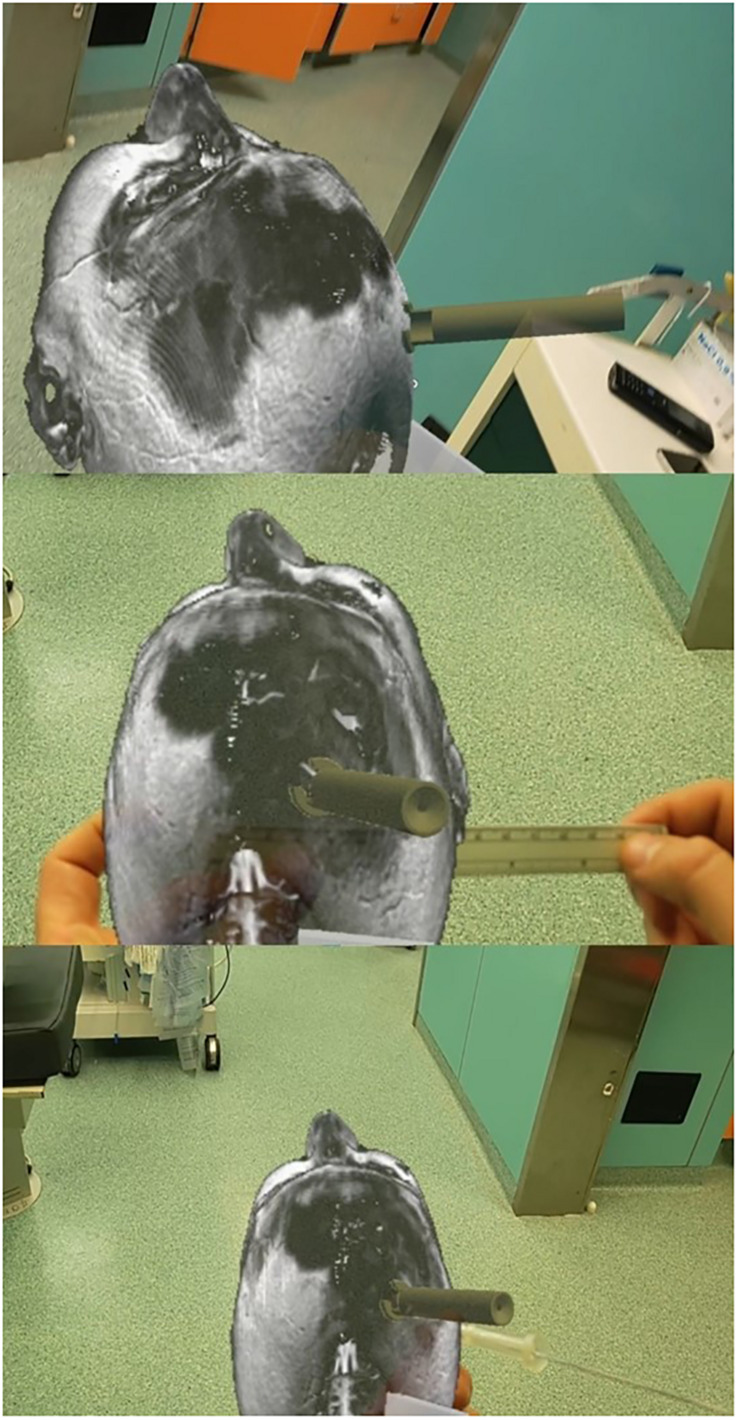
Augmented reality-based simulation (ApoQlar) to locate the entry point. A real ruler was overlapped on the hologram based on both MRI and CT scans; the actual device was overlapped over its holographic reconstruction, and the navigated stylet was introduced to improve familiarity with the procedure and technology.

We used Microsoft’s HoloLens mixed reality glasses and Virtual Surgery Intelligence (VSI) (ApoQlar, Hamburg, Germany). The model can be moved and “edited” in space via gesture control, and speech control can be used to record videos or take pictures. We uploaded the head CT scan data set and STL file of the device to reconstruct the patient’s anatomy and the holographic rendering of the device, respectively, to improve the immersive experience of the simulation (Figure 6).

#### 3D Neuronavigation-Based Simulation

We conducted a 3D neuronavigation-based simulation by performing a CT scan of the 3D plaster model. The data set obtained was merged with the patient’s head CT scan to allow visualization of the ventricles. The 3D model was then uploaded in BrainLab (Munich, Germany) planning station and used to simulate the introduction of the ventricular catheter in the plaster model, with the device overlapped on its holographic representation, verifying the correct trajectory navigating the catheter. The navigation could be associated with neuronavigation by using a navigated stylet or other navigation tools.

### Accuracy

**FIGURE 7:**
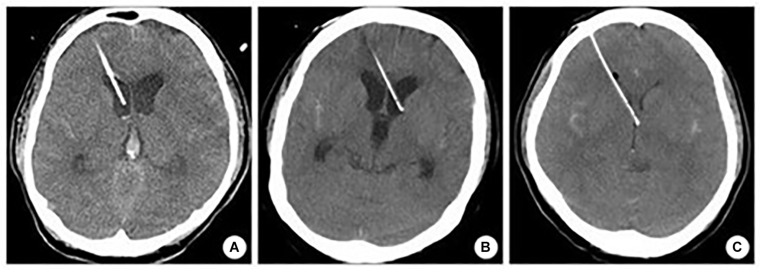
Kakarla grading system. **(A)** Homolateral lateral ventricle positioning. **(B)** Contralateral lateral ventricle positioning. **(C)** Misplacement.

The accuracy was assessed according to the Kakarla classification (Figure 7; Kakarla et al., 2008), which includes three grades (Table 1). The device’s accuracy has been proven to be equal to that of the neuronavigation because we applied the device according to craniometric parameters and using plaster models; furthermore, we applied the navigated stylet to confirm the accuracy of the trajectory provided by the guide. The 3D computer-based navigation should be considered a fine simulation step that geometrically demonstrates the mathematical reasons on which the device’s efficacy is based. The computer did not adjust the trajectory to bias the results and obtain the desired results; instead, it allowed to get the correct measures. The software allows to apply the device perpendicularly to the skull surface and, only after this step, to evaluate its trajectory.

**TABLE 1:** Kakarla grading system for ventricular catheter tip mispositioning.

**Grade**	**Placement accuracy**	**Catheter’s tip location**
**A**	Optimal/adequate	Ipsilateral frontal horn, including the tip of the third ventricle
**B**	Suboptimal (shallow)	Contralateral frontal horn
**C**	Suboptimal	Corpus callosum/interhemispheric fissure in eloquent tissue brainstem/cerebellum/internal capsule/basal ganglia/thalamus/occipital cortex/basal cisterns

We suggest considering this computer-based simulation step as the finest and most objective way to test the device in a simulation setting. In daily life, the head’s position can affect the accuracy of the EVD placement; thus, a guide that is of aid to get perpendicularity independently from the head position would be of great benefit.

## Results

The trajectory was assessed using an anonymized head CT scan of 25 patients (14 males and 11 females) with a median age of 71 (range 36–90) years. The mean of the Evans’ index was 0.28 (±0.03 standard deviation); see Table 2. The catheter tip distribution was calculated in 23/25 and 24/25 cases in the right and left hemispheres, respectively. The calculation was not performed in patients with a tumoral mass in the right hemisphere, and, in one case, the CT images did not include the entire skull.

**TABLE 2:** Computer-based simulation with report of Evans’ index, depth in both sides, and data analysis.

**Evans’ index**	**Positive R**	**Positive L**	**Depth R**	**Depth L**	**Age**	**Sex**	**Right X**	**Right Y**	**Left X**	**Left Y**
0.286	Yes	Yes	60	60	71	M	4.29	28.58	3.53	32.12
0.275	No	Yes	60	60	53	F	4.77	16.52	0	17.83
0.298	Yes	Yes	60	60	69	M	8.48	25.68	7.25	26.45
0.297	Yes	Yes	60	60	80	F	4.19	27.91	4.2	26.71
0.252	Yes	Yes	60	60	73	F	−1.64	14.95	0	14.91
0.334	Yes	Yes	60	60	73	M	7.66	16.61	1.74	17.9
0.237	Yes	Yes	60	60	85	F	5.9	18.68	3.83	20.23
0.328	Yes	Yes	60	60	82	F	7.97	26.65	7.95	20.4
0.314	Yes	Yes	60	60	68	M	8.56	22.6	3.64	25.79
0.289	Yes	Yes	50	60	71	M	11.46	26.19	2.52	32.37
0.26	Yes	Yes	60	60	59	F	3.54	20.98	2.83	22.48
0.321	Yes	Yes	60	60	76	M	7.32	24.8	9.38	28.67
0.277	Yes	Yes	60	60	83	F	11.67	24.36	5.25	26.5
0.306	Yes	Yes	60	60	78	M	11.45	31.62	8.78	36.24
0.309	Yes	Yes	60	60	64	M	6.47	25.67	1.38	27.39
0.267	Yes	Yes	60	60	63	F	3.65	29.26	4.51	28.06
0.293	Yes	Yes	60	60	78	M	9.86	28.35	9.64	27.78
0.259	Yes	Yes	60	60	36	F	6.91	22.7	1.55	25.91
0.319	Yes	Yes	60	60	50	M	3.66	29.97	−1.7	29.81
0.273	Yes	Yes	60	60	52	F	4.64	22.85	9.38	23.83
0.267	Yes	Yes	60	60	75	M	6.83	23.03	4.7	25.01
0.231	Yes	Yes	60	60	50	F	2.03	23.43	3.16	23.44
0.272	Yes	Yes	60	60	86	M	7.62	30.68	7.08	28.88

**FIGURE 8:**
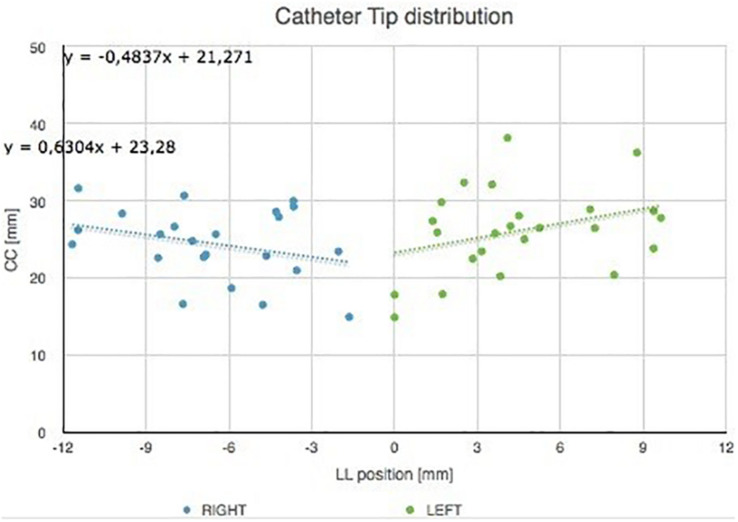
Catheter tip distribution in the craniocaudal (CC) and in the latero-lateral (LL) position in the left and right brain hemispheres, calculated in our cohort using the augmented reality tool. The trend lines of catheter tip position in the right and left lobes are indicated.

The catheter tip distribution in the craniocaudal (CC) and in the latero-lateral (LL) position, calculated in our cohort using the computer-based simulation in the left and right brain hemispheres, is shown in Figure 8.

**FIGURE 9:**
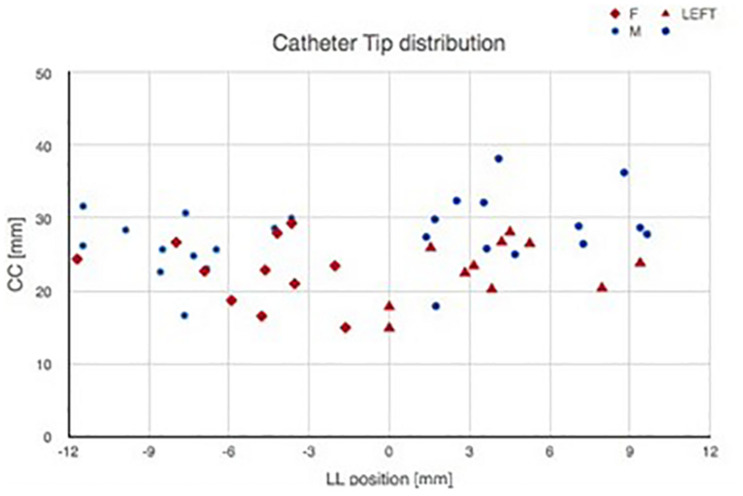
Catheter tip distribution against the patient sex/gender.

Figure 9 shows catheter tip distribution against patients’ sex/gender, highlighting, as expected, that CC position is lower for female than male patients due to skull shape and anatomical characteristics (*t*-test, *p* = 0.0003).

The cadaveric tests proved that any surgeon could use the device without any external help because it shows a good fixation inside the burr hole. The internal bone border created by the burr hole perforator, close to the dura, should be removed with a Kerrison rongeur to obtain the complete insertion of the proximal end of the device. The burr hole should have a minimum thickness of 5 mm for optimal insertion of the device. Thus, only in the case of a thick skull is it possible not to remove the inner skull layer and directly use the device.

The multimodal simulation performed with 3D neuronavigation and computer-based simulation showed Kakarla grade 1 in both sides after right and left EVD placements, in all cases but one, classified as grade 2. We considered only Kocher’s point, 11 cm from the nasion and 3 cm from the midline. The simulation with augmented reality, associated with 3D navigation using plaster skulls, demonstrated that the device positioned with the aid of mixed reality allowed an excellent entry point definition, as confirmed by the 3D navigation. The mean Evans’ index was 0.28 (Table 2).

### Geometric Considerations

**FIGURE 10:**
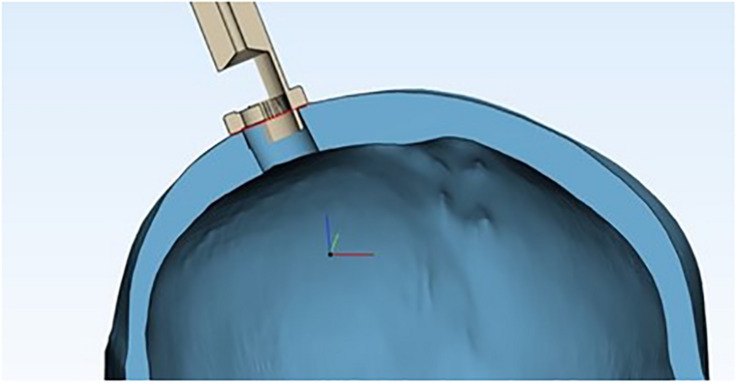
Cross section of the device’s proximal end inside the burr hole, showing the skull’s geometric properties, assuming the skull as a sphere and the burr hole as a chord.

The contact between the trocar and the skull occurs on a crown of points small enough to be assimilated to a circumference. Therefore, in order to study the geometric characteristics at the interface, we can analyze what happens in a generic section, as shown in Figure 10.

**FIGURE 11:**
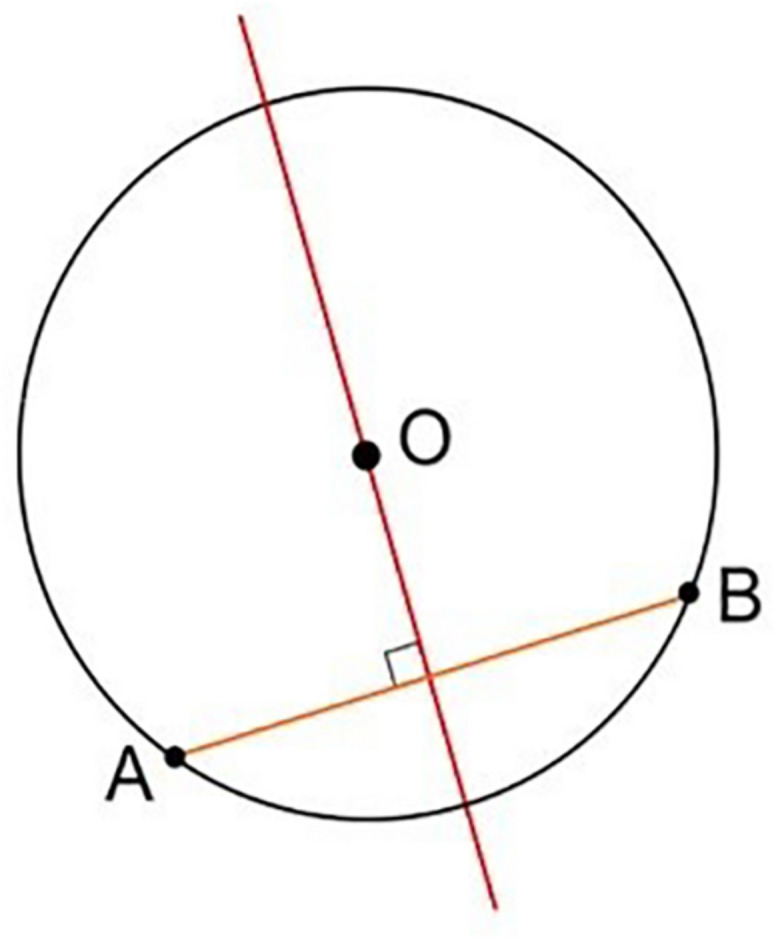
Geometric representation of the perpendicularity obtained, considering the plane as tangent to the spherical surface at the entry point.

The problem thus leads back to studying the geometric properties of circumference and chord (Figure 11).

**FIGURE 12:**
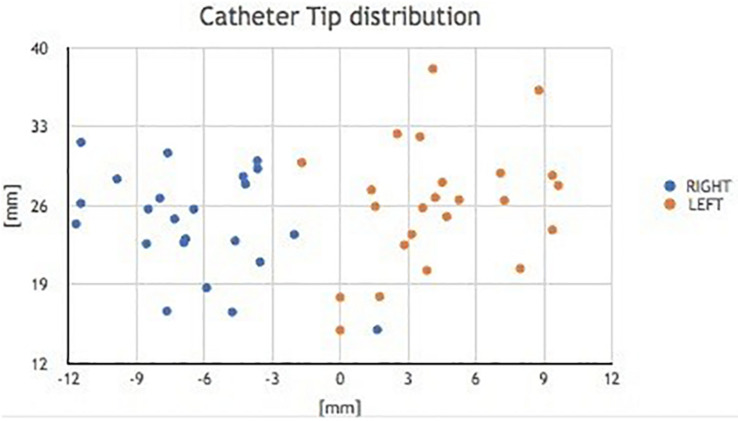
Catheter tip distribution to the right and left.

Contact occurs, for each section considered, at the extreme points of the chord (A and B in Figure 11). Its axis, perpendicular to the plane tangent to the spherical surface at the entry point, corresponds to the direction of entry of the catheter guided by the instrument (Figure 12).

## Discussion

### General Considerations

International recommendations for EVD placement suggest performing another burr hole if no CSF is obtained after more than three attempts (Greenberg, 2019), indirectly confirming that EVD misplacement is a risk that everybody will experience during their professional lives. The incidence of misplacement of the ventricular catheters reported in the literature is high, ranging from 12.3 to 60% according to the statistics (Saladino et al., 2009; Toma et al., 2009; Park et al., 2011; Bergdal et al., 2013; Chai et al., 2013; Rehman et al., 2013; Phillips et al., 2014), with possible associated complications (cerebral hematomas, neurological deficits, and seizures), which can dramatically worsen a patient’s clinical conditions (Woernle et al., 2011; Rosenbaum et al., 2013). Of notice, using the freehand technique, the only indirect sign of ventricular reach is the CSF drained from the distal catheter end, which does not give any information on the catheter’s trajectory.

Traversing brain parenchyma means creating damage from the cortex to the ventricle, with the risk of injury to cranial nerves, vessels, basal nuclei, or even the brainstem in case of mispositioning (Chai et al., 2013; Rosenbaum et al., 2013). Repeated attempts or even performing a second, contralateral, burr hole exponentially increases the risk of damage to eloquent structures.

We highlight the experimental nature of this study, in which we used merged technologies to assess the accuracy of the device. The use of the guide, together with the navigation, offered the possibility to assess the accuracy of the trajectory offered by the device, since CSF drain was not possible in a simulation setting.

### Device Technique

The present novel device aims to obtain the perpendicularity relative to the bone surface, thus reducing EVD misplacement rate without modifying the standard EVD placement method.

Once the device is introduced into the skull, the catheter will follow a perpendicular route to the ventricle without further trajectory adjustments, thus allowing EVD placement independently from the head’s position. The device’s utility includes the possibility to perform ventricular catheter placement with the same head rotation as used for ventriculoperitoneal shunt placement.

The device’s proximal end shows a notch that prevents excessive insertion, thus avoiding parenchymal lesion. Still, the essential function is represented by its geometric characteristic of a plane superimposed on the skull, which can be assimilated to a sphere, thus reproducing the tangent of a sphere, which in turn helps to achieve the needed perpendicularity. The critical point of the procedure is the correct selection of the burr hole, which must be performed at the desired location, 11 cm from the nasion and 3 cm from midline; of course, a wrong burr hole placement will alter the entire procedure, as what happens in the standard freehand technique. The novel guide is entirely non-invasive, so it does not add any injury to the patient. The burr hole angle does not affect the trajectory because the device works on the skull’s surface, and the internal bone border can be removed.

This preliminary, non-invasive investigation is expected to improve the learning curves of these procedures and decrease the rates of misplacement of the ventricular catheters by adopting a patient-specific approach. The process can be performed with no additional dose of ionizing radiation, just by using the conventional imaging acquired before the surgical procedure. The device can be used on its own but is neuronavigation-ready. Navigation is handy in the case of midline shifts, in which the normal anatomy is altered and it is not possible to use the typical anatomical landmarks and in the case of “slit ventricles,” in which the target is challenging to reach (Gil et al., 2002; Mahan et al., 2013; Jakola et al., 2014; Thomale et al., 2018). If used on a patient with no midline shift, the device is as accurate as navigation, which is then unnecessary, thus saving costs (Chai et al., 2013).

### Utility in Low- and Middle-Income Countries

The low economic impact is of great importance since this device could be particularly useful in low- and middle-income countries (LMICs), in which EVD placement is performed by non-neurosurgeons, mostly general surgeons, in remote areas where referral to neurosurgeons is time-consuming or impossible or the patients’ clinical conditions are worsening rapidly (Robertson et al., 2019a,b,c; Fuller et al., 2020). The aid of a simple guide for EVD placement could be of great benefit in those contexts, helping surgeons and improving outcomes. Given the high incidence of mispositioning among the neurosurgical statistics reported in the literature (Saladino et al., 2009; Toma et al., 2009; Park et al., 2011; Bergdal et al., 2013; Chai et al., 2013; Rehman et al., 2013; Phillips et al., 2014), the incidence of mispositioning in LMICs could be expected to be higher. The possibility of using a device that offers similar accuracy to neuronavigation could be of great benefit for LMICs, where expensive tools for neuronavigation are not available and non-neurosurgeons often perform EVD procedures in remote areas.

### Multimodal Simulation Tests

We did not consider midline shift because it includes an extensive range of conditions, causing the data to be non-homogeneous, and it is out of the interest of the study, which is focused on the assessment of the possibility to get the perpendicularity in the majority of cases in which EVD is required, i.e., patients affected by hydrocephalus, with no midline shift. Patients who require EVD placement should be distinguished from those requiring other surgeries, like patients affected by midline shift, in whom a craniotomy is usually needed. All this would have included biases and methodological inaccuracies that are not desirable and are not the core of this simulation study.

To obtain reliable results, we performed multimodal simulation by using the most advanced tools available. Plaster models of the skull were created to verify the accuracy of entry point definition by merging craniometric measures, augmented holographic reality reconstruction of the device, and 3D navigation. The latter was also used to assess the target by using a navigated stylet through the device, positioned inside the burr hole created in the plaster model. Augmented reality was also used to simulate the procedure using a holographic representation of both the skull and the device, thus improving familiarity with the simulation process and optimizing the device use. Of the utmost importance, the computer-based simulation using engineering software allows us to demonstrate the device’s accuracy mathematically.

According to our preliminary simulation study, the guide is a reliable device that deserves further investigation in larger cohorts and clinical settings. Considering a misplacement rate, reported in literature, of 12–60%, our results suggest that the device could improve safety and reduce the misplacement rate. Of course, clinical tests will provide more realistic data about its strength and limitations.

This study has been endorsed by the International Society of Reconstructive Neurosurgery (ISRN), and it has been proposed and accepted by five centers in Europe (Cannizzaro Hospital, Catania, Italy; Christian Doppler-Klinik – Universitätsklinikum der PMU, Salzburg, Austria; Fondazione Policlinico Universitario A. Gemelli IRCCS, Catholic University, Rome, Italy; Tor Vergata University Medical School, Rome, Italy; and Highly Specialized Hospital of National Importance Garibaldi, Catania, Italy), which could be further extended if needed.

Multimodal simulation offers unheard-of opportunities, allowing professionals to experience surgical procedures in a virtual setting that replicates, with high efficacy, the actual clinical practice, but with the further advantage of quantitatively analyzing all the steps, with the final aim to favor a safer and effective quality of care. Even in future clinical studies that we will promote, the association with neuronavigation will offer greater safety in EVD placement with this novel device.

Only after getting enough volume of data confirming the safety of EVD placement with this device would the procedure be performed only device-based with reasonable safety and efficacy. Again, this is a simulation, an experimental study to validate the trajectory offered by the device and its property to get the perpendicularity required immediately, indicated in literature to achieve the correct EVD placement.

## Conclusion

This study provides encouraging results on a simple, cheap device for EVD placement. Our multimodal simulation, through cadaveric, 3D computer-based simulation, 3D plaster modeling, 3D neuronavigation, and augmented reality, shows that the device could offer a safer and more effective EVD placement. Our results should be validated in a multicenter clinical study, based on the encouraging data obtained in the present simulation study.

## Patents

Number RM2014A000376: Guide for the positioning of cerebral catheters in neuronavigation, for the monitoring of intracranial pressure, CSF drainage in the case of sub-arachnoid hemorrhage or hydrocephalus; possible extension of drainage of abscesses and cerebral hematoma evacuation.

## Data Availability Statement

The original contributions presented in the study are included in the article/supplementary material, further inquiries can be directed to the corresponding author/s.

## Author Contributions

GU: conceptualization, writing—original draft preparation, supervision, and project administration. GU and LS: methodology. GS: methodology, form analysis, and writing—review and editing. RMi, SD, MG, and AS: software. SC, MV, DI, and GB: validation. GU, GS, LS, and KY: formal analysis and writing—review and editing. GU, MF, ST, GN, GR, and FG: investigation. GU, RMi, SD, and MG: resources. RMi, SD, and MG: data curation. GU, MS, and RMa: visualization. LS: funding acquisition. All authors have read and agreed to the published version of the manuscript.

## Conflict of Interest

Authors RMi, SD, and MG are employed by the company MtOrtho which does not produce the device. They simply used the software to obtain calculations. The remaining authors declare that the research was conducted in the absence of any commercial or financial relationships that could be construed as a potential conflict of interest.
